# An Ochered Fossil Marine Shell From the Mousterian of Fumane Cave, Italy

**DOI:** 10.1371/journal.pone.0068572

**Published:** 2013-07-10

**Authors:** Marco Peresani, Marian Vanhaeren, Ermanno Quaggiotto, Alain Queffelec, Francesco d’Errico

**Affiliations:** 1 Dipartimento di Studi Umanistici, Sezione di Preistoria e Antropologia, Università di Ferrara, Corso Ercole I d’Este 32, I-44100 Ferrara, Italy; 2 CNRS UMR 5199 PACEA-PPP, Université Bordeaux 1, Avenue des Facultés, F-33405 Talence, France; 3 Naturalistic-Archaeological Museum of Vicenza, Contrà S. Corona, 4, I-36100 Vicenza, Italy; 4 Department of Archaeology, Cultural and Religious Studies, University of Bergen, Postboks 7805, 5020 Bergen, Norway; University of Kansas, United States of America

## Abstract

A scanty but varied ensemble of finds challenges the idea that Neandertal material culture was essentially static and did not include symbolic items. In this study we report on a fragmentary Miocene-Pliocene fossil marine shell, *Aspa* *marginata* , discovered in a Discoid Mousterian layer of the Fumane Cave, northern Italy, dated to at least 47.6-45.0 Cal ky BP. The shell was collected by Neandertals at a fossil exposure probably located more than 100 kms from the site. Microscopic analysis of the shell surface identifies clusters of striations on the inner lip. A dark red substance, trapped inside micropits produced by bioeroders, is interpreted as pigment that was homogeneously smeared on the outer shell surface. Dispersive X-ray and Raman analysis identify the pigment as pure hematite. Of the four hypotheses we considered to explain the presence of this object at the site, two (tool, pigment container) are discarded because in contradiction with observations. Although the other two (“manuport”, personal ornament) are both possible, we favor the hypothesis that the object was modified and suspended by a ‘thread’ for visual display as a pendant. Together with contextual and chronometric data, our results support the hypothesis that deliberate transport and coloring of an exotic object, and perhaps its use as pendant, was a component of Neandertal symbolic culture, well before the earliest appearance of the anatomically modern humans in Europe.

## Introduction

Neandertal symbolic behavior is a controversial issue that has attracted much debate over the last thirty years [1,2,3,4,5,6,7,8]. Recent discoveries and reappraisals of ancient finds suggest that Neandertals were engaged in symbolically mediated behavior before the earliest appearance of anatomically modern humans in Europe. Burials of adults and children in and outside Europe are often considered the most striking evidence supporting the idea that intentional symbolic acts were part of Neandertal cultures [9,10,11]. Grave goods in the form of faunal remains, stone and bone tools, engraved bone, and rock slab engraved with cupules are reported at Neandertal primary burials from France and East Asia [12,10]. Rare objects such as crystals and fossils were apparently collected at Mousterian sites such as Combe Grenal and Chez Pourré-Chez-Comte [13,14]. Naturally perforated and ochered marine shells were recovered in Mousterian levels dated to ca 50 ky BP at Cueva de Los Aviones and Cueva Antón in the Iberian Peninsula [15]. Cave sites from Italy, France and Spain yielded evidence of intentional extraction of feathers [16,17] or terminal pedal phalanges of large raptors and other birds [18,19]. Use of pigment, as old as 200-250 ky BP [20], becomes widespread after 60 ky and is associated with the discovery of pigment processing tools and pigment containers [21,22,14,23]. This growing body of evidence creates a more dynamic image of Neandertal cultures and challenges the idea that they were essentially static, closed to innovation and without symbolic imaging.

Here we report on a fossil marine shell, *Aspa* *marginata* , discovered in a Mousterian layer (A9) of the Fumane Cave, northern Italy dated to 47.6 cal ky BP. We provide detailed information on the find and its context, investigate the potential sources of the fossil shell, document human modifications, and discuss its significance in the debate on the use of symbolic materials by Neandertals.

### THE ARCHAEOLOGICAL CONTEXT AND DATING

**Figure 1:**
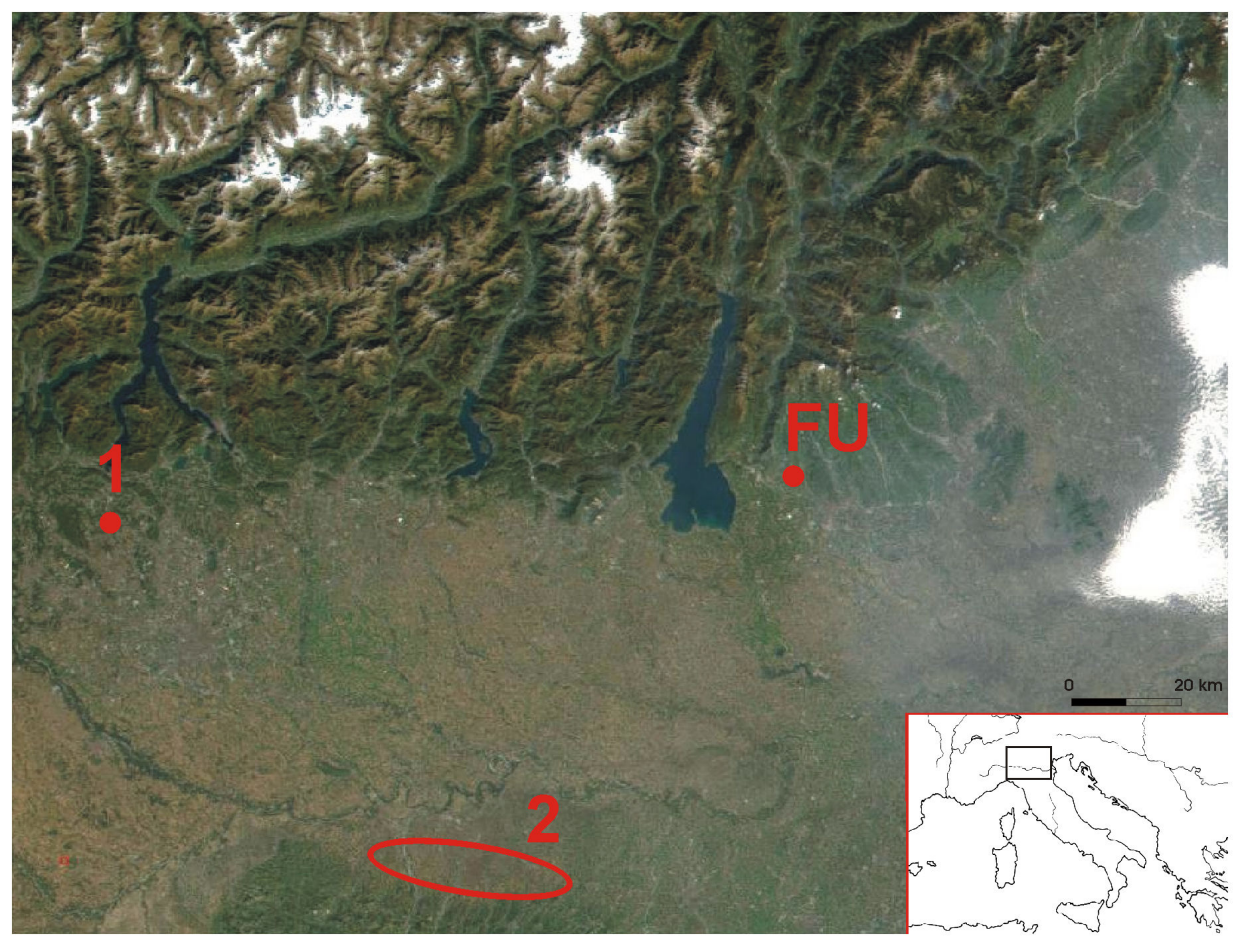
Location of Fumane cave (FU) and of fossil exposures with *Aspa* *marginata* shells. Cassina Rizzardi in the Lombardy Pre-Alps (1); Miocene and Pliocene exposures south of the Po Valley (2) (by www.visibleearth.nasa.gov).

Fumane Cave is located at the foot of the Venetian Pre-Alps in the western Lessini Mountains (Figure 1). The cave is part of a fossil karst system that formed in the Upper Lias oolitic sandstone [24] and is represented as a large entrance in which three tunnels, labelled A, B and C, converge (Figure S2). Excavations over the last two decades yielded a 2.5 m deep Late Middle and Early Upper Paleolithic sequence sealed by thick slope waste deposits (Figure S1).

The Middle Paleolithic deposits consist of numerous thin to very thin parallel levels and lenses, slightly tilted towards the outside of the cave, which are grouped into nine stratigraphic units labelled from bottom to top A13 to A5 (Figure S2). The lowermost units A13 and A12 are archaeologically sterile and characterized by flat angular stones embedded in sandy or loamy matrix. Units A11 and A10 yielded anthropogenic lenses with abundant lithic artifacts and faunal remains embedded in various levels of stones resulting from frost-shattering and characterized by a variable incidence of a loamy fine fraction. Unit A9 shows a succession of frost-shattered loose breccia, aeolian silts and sands, and dark anthropogenic sediments with archaeological remains. Unit A8, only present in an area outside the cave [25], is considered to be a facies of unit A9. This latter unit is sealed by unit A7, which is composed of stones and light brown silt. Although mainly sterile, unit A7 contains some reworked lithic artefacts and bone remains in the cave entrance area where it is affected by cryoturbation. Overlying unit A6 is a dark sediment with a high content of anthropogenic remains. Unit A5 is composed of loose stones with a loamy fine fraction and few archaeological remains.

The Upper Paleolithic sequence includes six stratigraphic units labelled from bottom to top A4 to A1, D6 and D3. The bottom units A4 and A3 consist of frost-shattered slabs with variable sand content and aeolian dust that becomes more prevalent toward the outermost part of the cave. Dwelling structures with hearths and a toss zone have been identified as well as numerous lithic artifacts and bones. Unit A2 displays intense anthropogenic sedimentation, toss zones, combustion structures, and two large zones of reddened sediment due to abundant ochre. One small zone lies at the top of the unit, at the entrance of the cave, the second is larger and continues towards the rear of the cave, at the base of unit A2 (layer A2R [26]). Unit A1 is a thin level exclusively present in the area at the entrance of the cave. Units D6 and D3 are thick levels made of large stone blocks, sands, aeolian dust with limited evidence of human occupation.

Lithic artefacts from the Middle Paleolithic sequence (Peresani 2012) belong to a Levallois Mousterian for the lower (A11, A10) and upper (A6-A5) units and to a Discoid Mousterian for the middle units (A9, A8). Starting from the bottom (A11), the abundance of lithics show that Levallois technology was used to produce a large amount of flakes, cores and retouched tools. Above this lowest layer, across the set of levels of unit A10, the lithics are attributed to the Levallois and Discoid reduction sequences, either alternating or coexisting in the same level. The Discoid industry becomes exclusive in units A9 and A8 [28] where it is typically represented by thick flakes, pseudo-Levallois points, backed flakes with a thin opposite edge, polygonal and triangular flakes and few scrapers, points and denticulates. Layer 7 is a sterile level, but unit A6 yielded a Levallois assemblage associated with sporadic artifacts attributed to other flaking methods [29]. The few artifacts from unit A5 also belong to a Levallois industry.

Analysis of the faunal remains in the Mousterian levels shows that red deer (*Cervus*), roe deer ( *Capreolus* ), ibex (*Capra*), chamois (*Rupicapra*) and giant deer (*Megalocerus*), were the most hunted species in the Mousterian [18,30,31]. In addition, exploitation of brown bear (*Ursus*) and fox (*Vulpes*) is found in unit A6 and A5 [32]. The large and varied avifauna from these same units reveals unusual human modifications on species that are not clearly related to consumption or utilitarian purposes [30].

Stone tools from the Upper Paleolithic sequence are attributed to the Uluzzian technocomplex for units A4 and A3 [33] and to the Proto-Aurignacian for units A2-A1 and D6-D3 [34]. Faunal remains from units A4 and A3 reveal exploitation of red deer, ibex and carnivores [35], while ibex dominates the faunal assemblage from units A2-A1, D6 and D3 [36]. Dwelling structures, bone and antler tools, painted stones and pierced mollusc shells are found in the Proto-Aurignacian units [37,34,26]. Pierced shells belong to 60 taxa, 53 of which belong to the class of Gastropoda, 6 of Bivalvia and 1 of Scaphopoda, *Homalopoma* *sanguineum* being the most represented taxon. Direct AMS dating of perforated shells belonging to *Homalopoma* *sanguineum* *,* *Nassarius* *circumcinctus* , and *Glycymerisinsubrica* are consistent with other ^14^C ages obtained from the Proto-Aurignacian units and demonstrate that they were gathered on contemporaneous beaches [38]. Use wear on well preserved perforation edges indicate that the shells were used as personal ornament [39].

Bayesian analysis of radiocarbon ages from the Fumane sequence suggests that the Proto-Aurignacian units accumulated between 40.5 and 41.9 ky cal BP, the Uluzzian between 41.9 and 43.9 ky cal BP, the final Mousterian units A5 and A6 between 43.9 and 44.8 ky cal BP and underlying Mousterian units A8 to A11 between 44.8 and 47.6 ky cal BP [40,41].

### THE FIND CONTEXT

**Figure 2:**
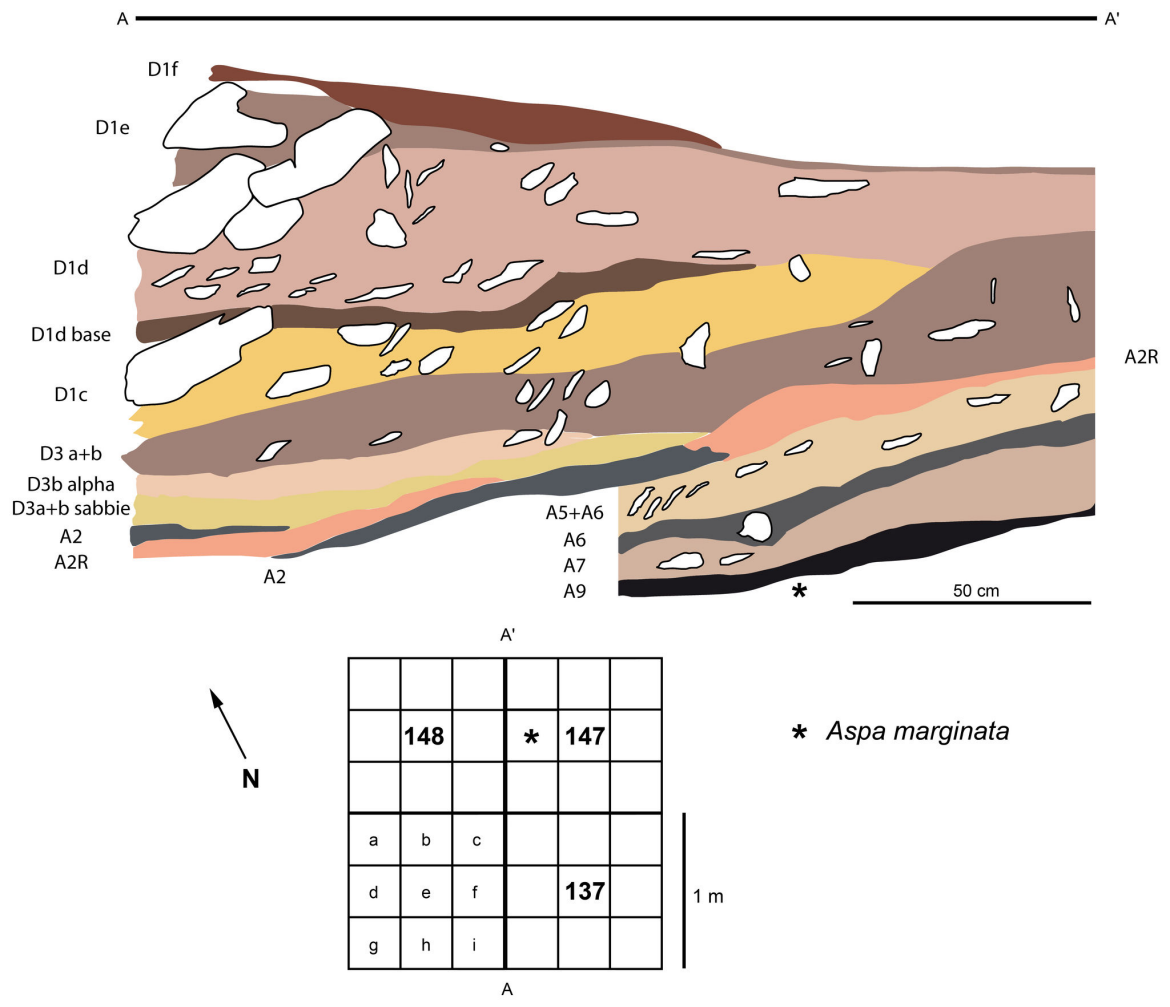
Stratigraphy of the Fumane Cave sequence in squares 137-147.

The fossil marine shell was discovered during the 2005 excavations, in unit A9, subsquare 147d, in an area located at the back of the cave, 7m beyond the present-day drip-line (Figures S2 and S3>). Excavated over a surface of 68 m^2^, Unit A9 was in this area overlain by units D3 (15-25 cm), A2 (3-10 cm), A5+A6 (13 cm), A6 (5 cm), and A7 (10-15 cm) (Figure 2). In sub-square 147d, Unit A9 was 15 cm thick and subdivided into two sublevels A9 and A9base the latter of which was richer in archaeological remains. No traces of bioturbation, cryoturbation or injections of allocthonous sediment were recorded during excavation of unit A9. No artefacts diagnostic of the overlying and underlying Levallois Mousterian units A6 and A10 respectively were identified in Unit A9. The same holds for the Uluzzian and Proto-Aurignacian artefacts, which were absent from the stratum. The associated faunal remains are dominated by cervids (*Cervus, Megalocerus* and *Capreolus* ), followed by bovids and caprids ( *Rupicapra* and *Ibex*). Hunting focused on adult and old individuals [31]. Six ^14^C and one ESR radiometric determinations (Table S1) are available for Unit A9 [40]. The former range between 36,450±400 ^14^C BP (LTL-573A) and 42,750±700 ^14^C BP (LTL-376A); the latter is 46,000±7,000 (FU-0004). Considering the ^14^C ages obtained from overlying units, known biases due to sample contamination in this age range, and the ESR result, it is probable that the oldest ^14^C age (47.6 ky cal BP) is the most reliable minimum age for Unit A9.

### THE SHELL

#### Taxonomy and provenance

**Figure 3:**
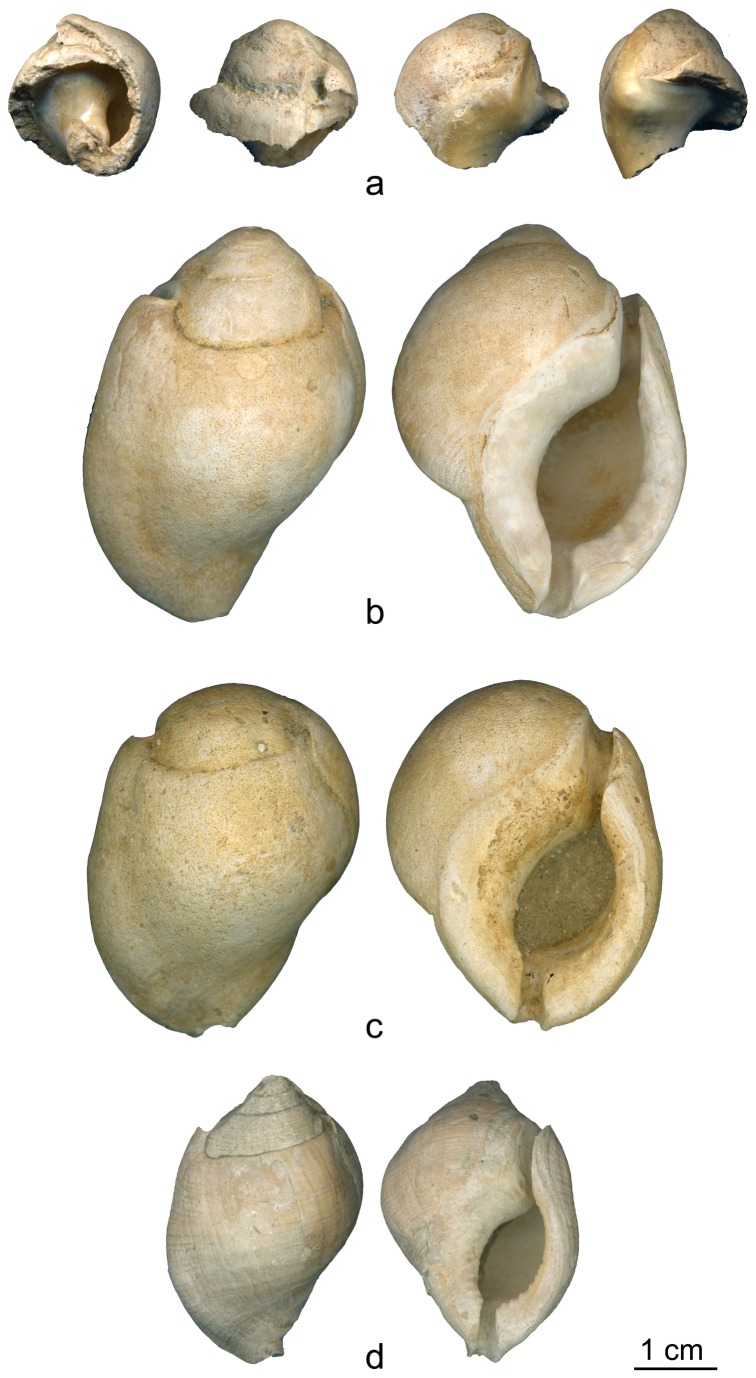
*Aspa* *marginata* shells. The broken *Aspa* *marginata* shell (a) from the Mousterian stratigraphic Unit A9 of Fumane Cave and three complete natural fossil shells (b–d) of the same species from Pliocene deposits close to Asti, Piemonte region, Italy.

The object (Figure 3a) is an apical fragment of a thick gastropod shell with a smooth surface, a blunt short spire and a deep siphonal canal. These features are characteristic of the Bursidae species *Aspa* *marginata* (Gmelin, 1791). This species is common in the European Miocene Paratethyan fossil assemblages and well-known from the Italian Pliocene fossil record [43,44,45,46,47,48]. The shell cannot derive from the Early Jurassic Formation in which Fumane cave is situated. Pleistocene to recent representatives of this species are restricted to the North-western African coast [48] from southern Spain to Angola [49], Madeira [50] and the Canary [51] and Cape Verde Islands [52,53]. It is most likely that the *Aspa* *marginata* from Fumane Cave was collected by Neandertals at a fossil exposure at more than 110 kilometers from the site. The closest fossil *Aspa* *marginata* shells are reported from Miocene and Pliocene exposures south of the Po Valley [54,43,55,56,48,57]. Pliocene exposures or reworked invertebrate remains are also occasionally found in the Veneto region near Cornuda and Anzano di Vittorio Veneto [58,59,60,24] and Lombardy region near Taino, Val Faido, Folla di Induno, Pontegana, Cassina Rizzardi, Almenno, Nese, Castenedolo [61,62,63]. Although *Aspa* *marginata* could be theoretically found at these sites, the species is only reported from one of them, Cassina Rizzardi in the Lombardy Pre-Alps [61,62] (Figure 1).

#### Morphometric, taphonomic, and chemical analyses

**Figure 4:**
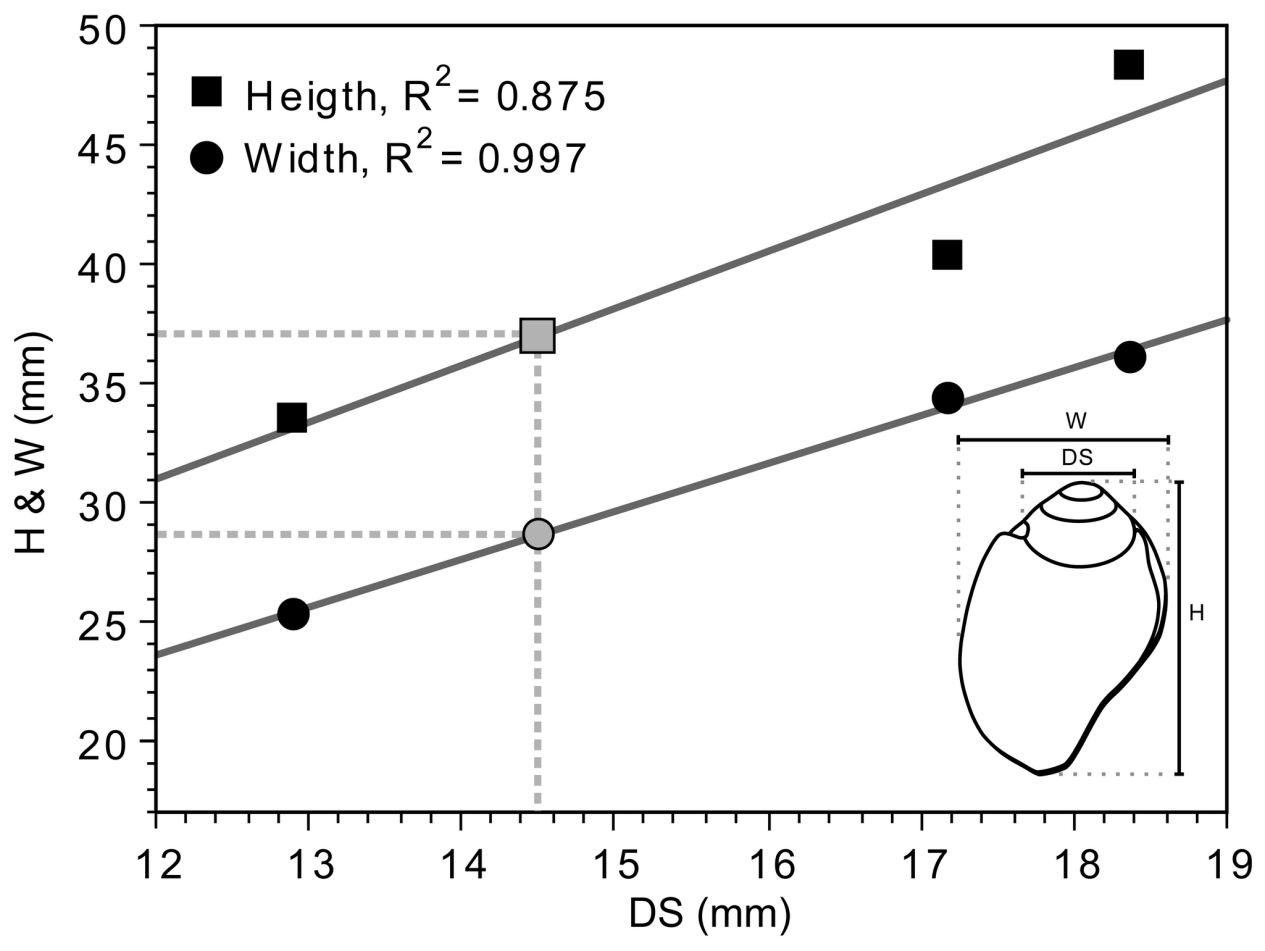
Estimation of the original size of the broken *Aspa* *marginata* from Fumane. Estimation (gray symbols) of the original height (H) and width (W) have been obtained by correlating the diameter of the last body whorl suture (DS) with the height (black squares) and width (black dots) of three fossil specimens from Pliocene deposits close to Asti, Piemonte region.

**Figure 5:**
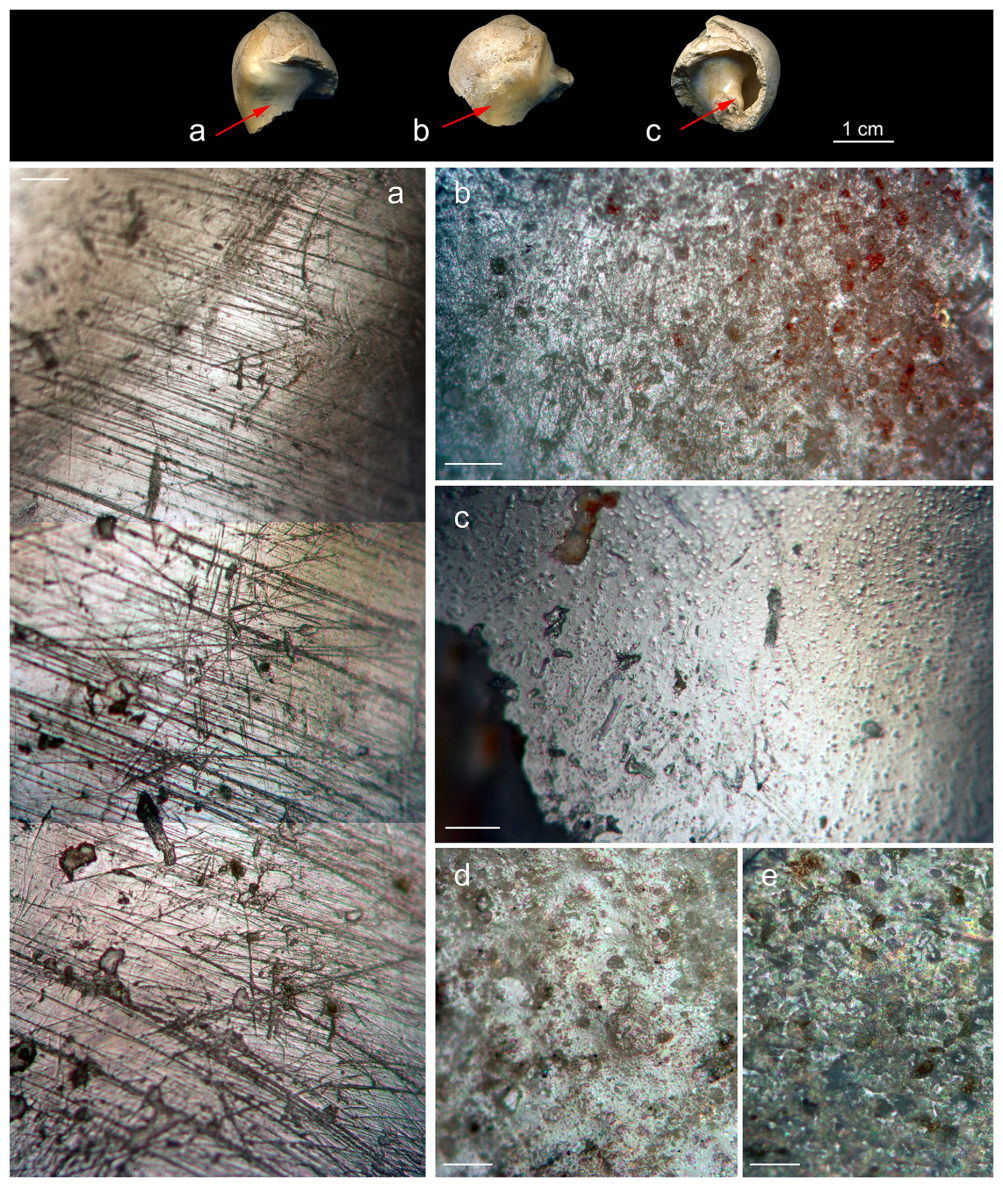
Details of the surface of *Aspa* *marginata* shells. Above, location of the micrographs (a–c) taken on the Fumane *Aspa* *marginata* ; d-e: inner lips of two *Aspa* *marginata* from Pliocene deposits close to Asti, Piemonte region. Notice in *b* the palympsest of striations present on the inner lip of the archaeological specimen. Scales = 100 µm unless indicated otherwise.

**Figure 6:**
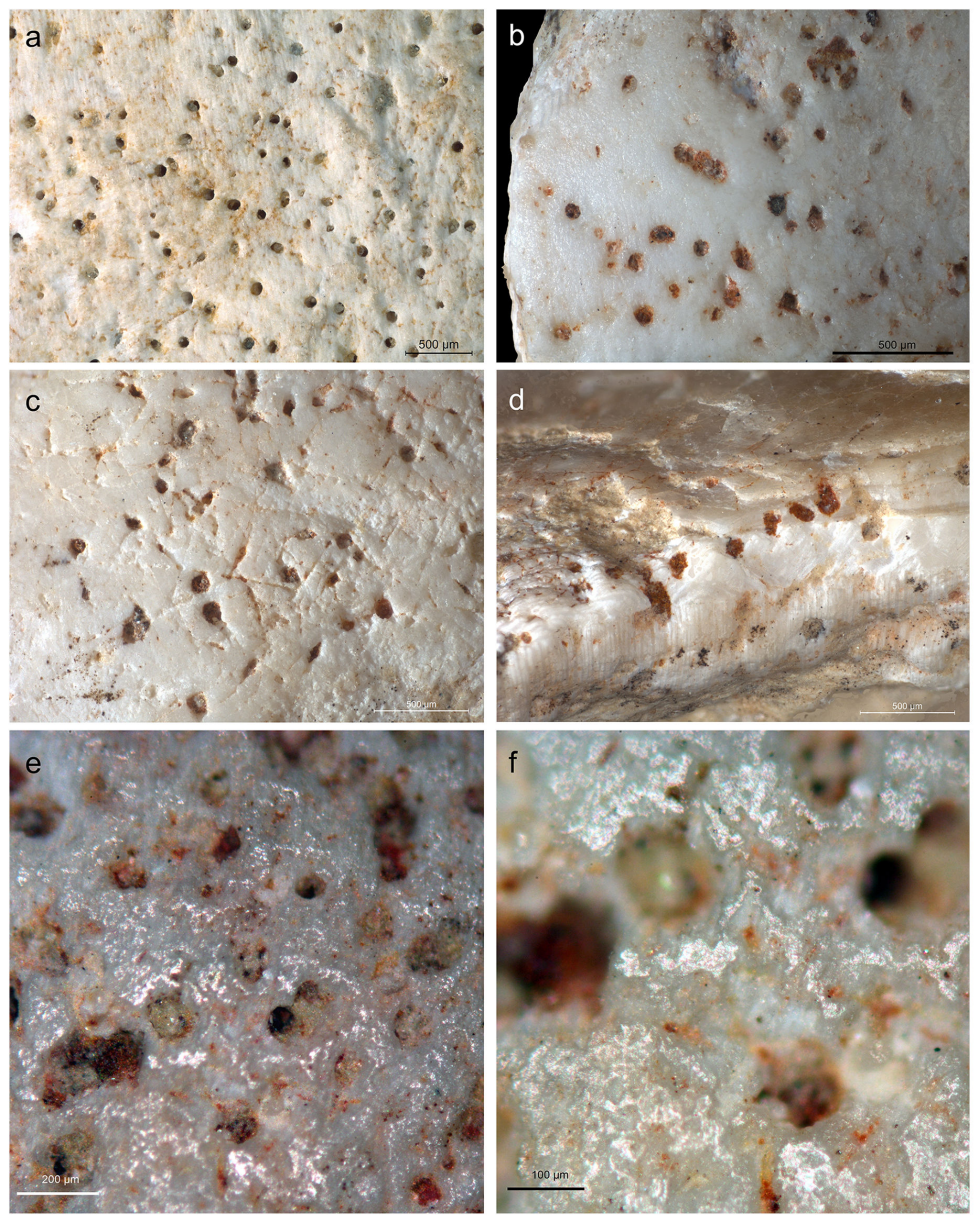
Outer surface of an *Aspa* *marginata* shell from the reference collection (a, and from the Fumane specimen (b–f). Notice the presence on both shells of pits produced by bioeroders associated, at Fumane, with networks of micro-grooves (c) due to the same taphonomic agent. All micro-concavities on the Fumane specimen (b–f), including pits truncated by the shell fracture (d) are filled with a red substance and prominent areas are affected by a slight polish (e–f).

The shell is beige in color and bears an old, irregular breakage removing most of the last spiral whorl. The original size of the specimen is estimated at about 34 mm in height and 24 mm in width by correlating the diameter of the body whorl suture with the height and width of three fossil specimens from Pliocene deposits close to Asti, Piemonte region (Table 1 Figures 3b–d, 4). Microscopic analysis of the shell surface reveals an area on the inner lip, close to the posterior canal, covered with clusters of striations. Between 1 and 10 µm wide, these striations are oriented perpendicular to the shell main axis (Figure 5a). They are absent on the remainder of the shell surface (Figures 5b-c, 6e-f) and on *Aspa* *marginata* from the reference collection (Figure 5d-e). These clusters of striations were likely produced by abrasive particles incorporated in a medium that has repeatedly rubbed a distinct area of the inner lip.

**Table 1:** Morphometric data on the *Aspa* *marginata* shell from the Mousterian stratigraphic Unit A9 of Fumane Cave and on complete natural fossil shells from a malacological reference collection.

** *Aspa* *marginata* **	**Height**	**Width**		**Width spire**
**shell**	**(mm)**	**(mm)**		**(mm)**
Fumane*				14.67
Asti**	34.83	23.70		13.00
Asti**	42.74	34.07		19.23
Asti**	48.26	35.89		21.05

*archaeological

**malacological reference collection

The shell’s outer surface is covered with micropits and, occasionally, networks of grooves produced by bioeroders that altered the shell during the life or shortly after the death of the mollusk (Figure 6b-c, e-f). These pits and grooves are filled with a dark red substance. Microscopic residues of this substance are also trapped inside irregularities of the shell surface (Figure 6 e-f). This red substance is absent on the prominent areas of the shell microtopography, which display a slight polish (Figure 6e-f), and virtually absent inside the shell and on the shell fracture (Figure 6d). Analysis of the fracture identifies truncated pits still filled with the red substance (Figure 6d). The above features suggest that the red substance was originally more abundant on the shell surface before being partially erased by a gentle post-depositional abrasion.

**Figure 7:**
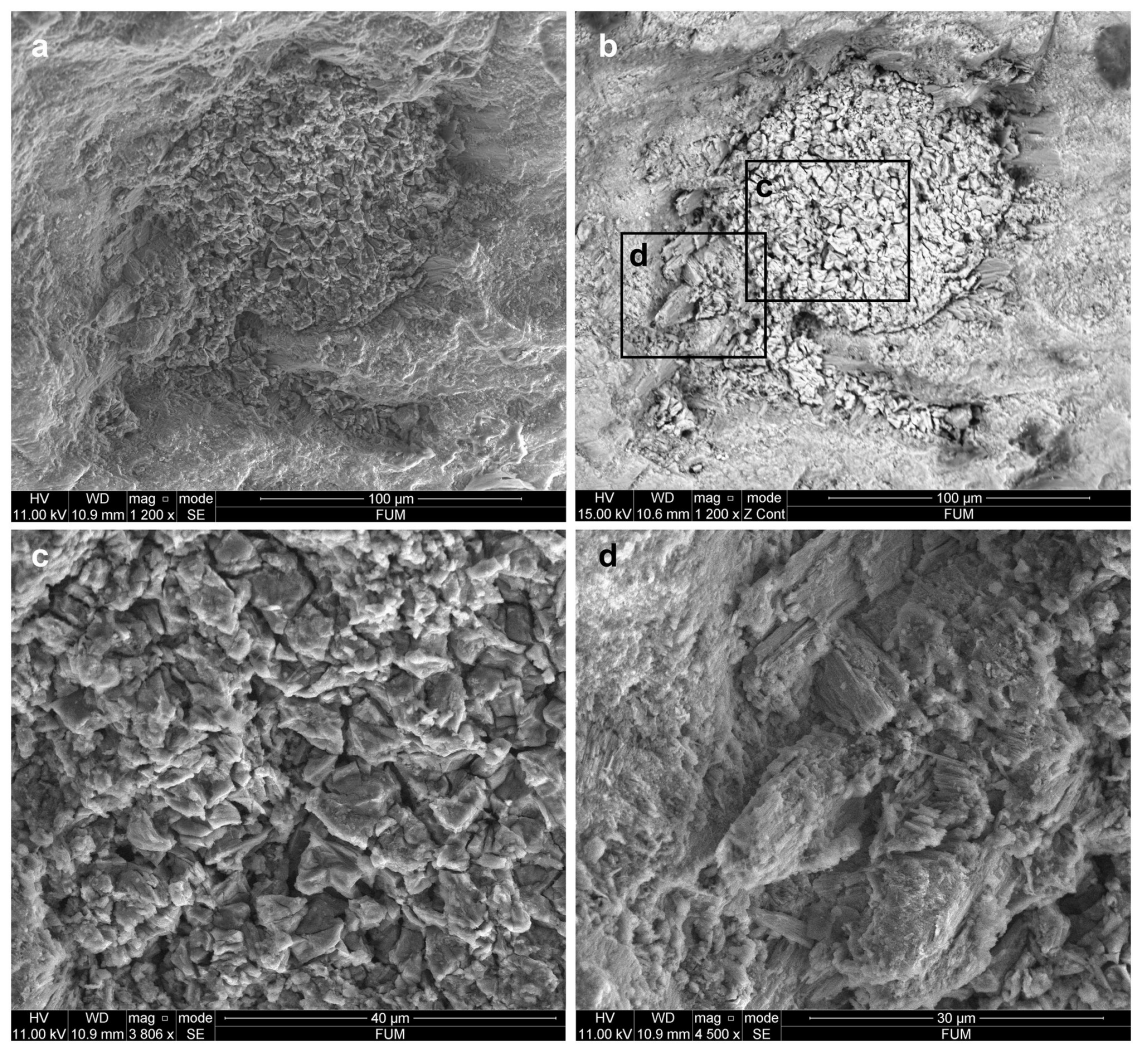
Scanning Electron Microscope micrographs. Micrographs have been obtained in secondary (a, c, d) and back-scattered electron detector mode (b) of a micropit filled with red substance on the Fumane *Aspa* *marginata* shell (see text).

**Figure 8:**
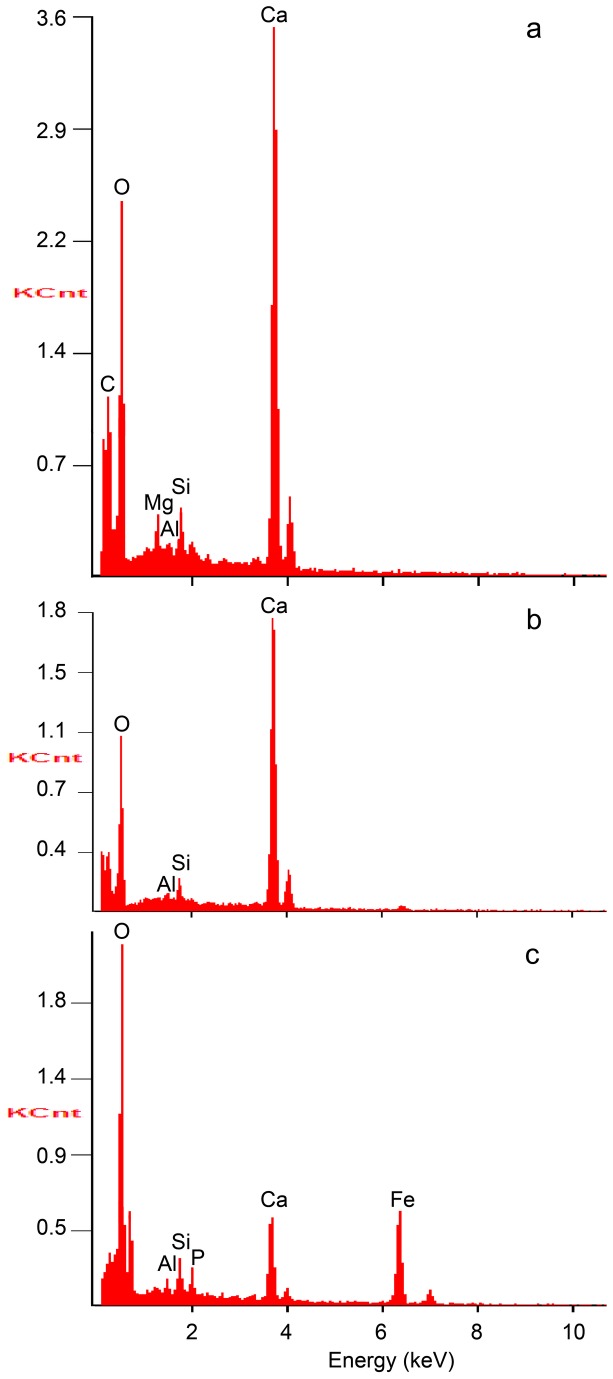
Results of the Energy Dispersive X-ray analysis performed on the Fumane *Aspa* *marginata* shell. a: outer shell surface, b: broken crystals on the edge of the micropit shown in Figure 7d, c: red substance filling the pit (Figure 7c).

**Figure 9:**
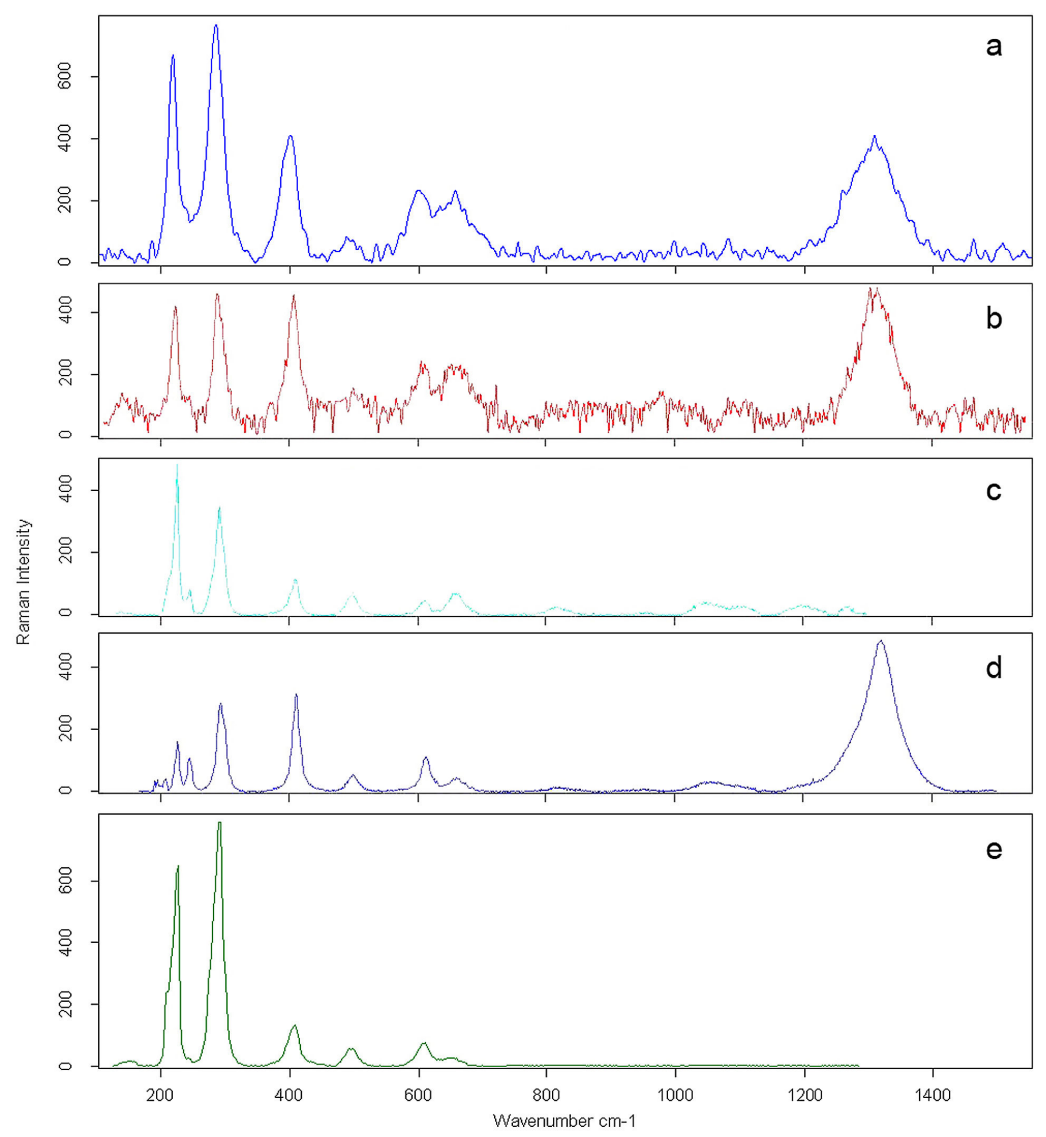
Comparison of Raman spectra. Raman spectra of the red substance trapped in two different pits on the surface of the Fumane *Aspa* *marginata* shell (a, b) and reference spectra for hematite (c, d, e) from the Rruff database [99].

Scanning electron microscope analysis of the red substance trapped in a pit reveals a homogeneous, amorphous matter overrun with microcracks, composed of heavy chemical elements (Figure 7a–c). The pit edge displays broken elongated crystals (Figure 7b, d), identified by Energy Dispersive X-ray analysis (EDX) as calcium carbonate (calcite or aragonite), i.e. shell fragments (Figure 8a-b Table 2). This pattern suggests that the infilling substance entered the pit forcefully and broke in the process with calcium carbonate crystals located at the periphery of the pits. EDX analysis identifies the red substance as a pure iron oxide associated with Ca and trivial proportions of Si, P and Al (Figure 8c Table 2). Raman analysis identifies this iron oxide as hematite (Fe2O3) (Figure 9).

**Table 2:** Mass percentage of chemical elements detected by Energy Dispersive X-ray analysis on selected spots of the Fumane *Aspa* *marginata* .

		**Spot**		
	**a**	**b**		**c**
**Element**	(Wt%)	(Wt%)		(Wt%)
**C**	12.23
**O**	42.01	46.71		37.81
**Mg**	1.73
**Al**	0.41	0.55		1.02
**Si**	1.93	1.81		3.00
**P**				1.98
**Ca**	41.70	50.32		11.99
**Fe**				44.19

a: outer shell surface, b: broken crystals on the edge of the micropit shown in Figure 7d, c: red substance filling the pit (Figure 7c).

## Discussion and Conclusion

Different hypotheses can be proposed to account for the presence of the *Aspa* *marginata* shell in the Mousterian unit A9 of Fumane Cave. The possibility that it results from percolation through the overlying Upper and Middle Paleolithic layers must be rejected for five reasons. First, contrary to other areas of the site [25], there are no sedimentary signatures of post-depositional mixing, be it cryoturbation, bioturbation, burrowing or other deformations of the stratigraphy, between stratigraphic units in the area where the shell was found. Second, unit A9 is separated from the overlying Upper and Middle Paleolithic units by a thick compact, continuous sterile unit A7. Third, no Upper Paleolithic cultural material of any type or size has been recovered in any of the Mousterian levels. Small sized elements, including tiny gastropod shells and minute fragments of Dufour bladelets, are numerous in the Aurignacian units, including in the area in strata above where the *Aspa* *marginata* was found, and could have percolated down if such processes occurred. Fourth, all units have yielded lithic assemblages displaying consistent technological features diagnostic of distinct technocomplexes. Fifth, the absence of *Aspa* *marginata* from the more than 800 shell beads present in the Aurignacian units further demonstrates that the shell cannot come from these units and supports its attribution to Mousterian unit A9.

It remains, then, how to explain the occurrence of a unique ochered fossil shell in this unit? There seems to be no alternative but that its presence in unit A9 and ochre staining is result from human agency. Results have shown that the *Aspa* *marginata* shell cannot come from the Fumane Cave wall and must have been collected by Mousterians at a Miocene or Pliocene fossil outcrop. While future research may establish another exposure site with *Aspa* *marginata* closer to the site, for now a review of potential sources shows that the closest exposure sites are located some 110 km south-west of the Fumane Cave. The Miocene and Pliocene formations in which fossil *Aspa* *marginata* occurs are generally composed of a silty-clay with sand and poor iron content, which significantly differs from the clay/colloidal appearance of the iron rich compound trapped in the pits and grooves of the archaeological shell (Figure 7c). This suggests that red pigment was not present on the fossil shell when it was collected by Neandertals. Red ochre of comparable elemental composition is used to paint the slabs found in Fumane’s Aurignacian unit A2 [64] and found in the form of lumps in the same layer [65]. While it is found in superior levels, the ochre detected on the *Aspa* *marginata* shell is unlikely a result from post-depositional percolation from overlying ochre rich Aurignacian unit A2R. If ochre would have percolated one would expect to find red pigment staining on artifacts and sediment in intermediate units A7, A6, A5+A6, which is not the case. Although the precise provenance of the pigment used in the Aurignacian unit is uncertain, karst fissures in Lower Jurassic formations filled with hematite rich deposits are known at a number of quarries located between 5 and 20 km far from the site [66]. These sources may have been used by Neandertals to collect the high quality iron oxides trapped into concavities of the shell surface.

Four hypotheses can be proposed to refine our interpretation of this find. The shell may have been used as a tool, a pigment container, a “manuport”, or a personal ornament. By manuport we mean a natural object unmodified or very marginally modified and moved from its original context by human agency and later curated, deposited, lost or discarded at an archaeological site. *Callista* *chione* and *Glycymeris* sp. bivalve shells were modified by breakage and retouch by Neandertals at a number of Italian and Greek sites [67]. In our case, however, the absence of traces of modification or use that may support the involvement of the *Aspa* *marginata* in a functional activity runs counter to the interpretation that it functioned as a tool. Ochre may be present in shells used as storage devices [68]. In the case of the *Aspa* *marginata* , however, the hypothesis of a pigment container is not concordant with the limited capacity of this shell for storage, comparative to its weight, and the absence of pigment inside the shell. Ochre may be used in adhesives [69], but neither pigment residues nor traces of other mineral or organic additives were detected on the Discoid lithic production from unit A9 [70]. Evidence that the pigment was homogeneously smeared on the outer shell surface also goes against the hypothesis of a use as pigment container since it supports the idea that the red staining results from a deliberate action and must have been more intense in the past. “Manuports” from Lower, Middle and Upper Paleolithic sites from Africa and Asia have been interpreted in a number of different ways. Exogenous pieces of stone raw material from Olduvai [71,72], considered as missiles by Isaac [73], Cannell [74], Calvin [75] and Bingham [76] have been recently re-interpreted as ecofacts by de la Torre and Mora [77].

A few Lower and Middle Paleolithic sites preserve exotic objects with no obvious functional role and striking visual appearance such as quartz crystals, fossils, shells, and natural objects mimicking human or animal shapes [78,79,80,81,82,83,84,85,86,87,88,89]. These are interpreted as the first evidence for the ability to distinguish ordinary from exotic items, to create conscious cultural taxonomies, and/or to detect iconicity in the natural world. Some argue these sporadic finds would have prompted the mental bridge between referent and referrer thus igniting the creation of symbolic material cultures [86,83,87]. Although this possibility cannot be discarded, three reasons may favor the interpretation of the *Aspa* *marginata* from Fumane as a pendant, i.e. an object conceived to be suspended for visual display body through threading or stringing. The attention put to uniformly cover the outer shell surface with good quality red pigment suggests that this action may have been performed to make the object suitable for visual display. The wear detected on the inner lip, made of overlapping groups of striations oriented perpendicular to the shell main axis, is consistent with a sustained friction produced by a cord rich in abrasive particles, such as sinew. The absence of pigment on the shell fracture is most consistent with this item being used as a pendant.

In conclusion, analysis of the *Aspa* *marginata* found in Fumane Unit 9 shows that this fossil gastropod was collected by Neandertals, makers of the Discoid industry, at a Miocene or Pliocene fossil outcrop, the closest of which is located more than one hundred kilometers from the site. The shell was smeared with a pure, finely ground, hematite powder, probably mixed with a liquid. It was perhaps perforated and used as a personal ornament before being discarded, lost or intentionally left at Fumane Cave, some 47,6-45,0 cal ky BP. The minimum age of the Fumane unit in which the *Aspa* *marginata* was found predates the oldest available dates for the arrival of anatomically modern humans (AMH) in Europe [90] thus supporting the hypothesis that deliberate transport and coloring of exotic objects, and perhaps their use as pendants, was a component of Neandertal cultures [91,92,15]. That the pendant appears well before the presumed first appearance of AMH in Europe [93, but see 90,94] indicates that Neandertals made this art object without the influence of AMH. The use of this shell by Neandertals as a result of contact with immigrant AMH is also contradicted by the absence of this particular taxon of shell at Early Upper Paleolithic sites across Europe [95,96]. The only other Paleolithic occurrence is a specimen found in the Epigravettian horizons of Riparo Tagliente in the Lessini Mountains of NE Italy [97]. Thus, this discovery adds to the ever-increasing evidence that Neandertals had symbolic items as part of their culture. Future discoveries will only add to our appreciation of Neandertals shared capacities with us.

## Materials and Methods

### Ethics Statement

All necessary permits were obtained from the Italian Ministery of Culture for the described study, which complied with all relevant regulations.

The unique identification number of the specimen analysed is VR09356.

I confirm that the person concerned in Figure S3 has seen this manuscript and figure and has provided written informed consent, as outlined in the PLOS consent form, to publication of his photograph.

Repository information: the specimen is temporary housed at the University of Ferrara, in the Section of Prehistory and Anthropology, Corso Ercole I d’Este Ferrara, with the permission of the Ministry of Culture - Veneto Archaeological Superintendence.

The archaeological deposits were systematically excavated within 33x33cm subsquares. All ≥5cm complete or fragmented lithics, bones, teeth and identifiable faunal fragments of ≤5cm were 3D plotted. Smaller remains were recovered from 2x2mm wet sieving and attributed subsquares and sub-units.

Taxonomic identification of *Aspa* *marginata* (Gmelin, 1791), its synonyms, past and present day geographical distribution and supraspecific systematic position was made by reference to Beu [48] and the World Register of Marine Species [98]. To establish the stratigraphic and geographic distribution of fossil *Aspa* *marginata* , we searched relevant geological and paleontological literature with special emphasis on the Miocene and Pliocene fauna in the north of Italy. Microscopic images were acquired with a motorized Leica Z6 APOA microscope equipped with a DFC420 digital camera and a Leica Application Suite with the Multifocus module. High magnification images were acquired with a reflected light Leica DM 2500M microscope. Secondary and back-scattered electron detector mode scanning electron microscopy images, as well as Energy Dispersive X-ray analysis were performed using a Quanta 200 scanning electron microscope with a voltage of 25 Kv. Raman spectra were obtained with a confocal microspectrometer SENTERRA (Bruker Optics, Ettlingen, Germany) equipped with a 532 nm exciting line (spectra acquired with 2 mW laser power, 20 coadditions of 10 s excitation with a 50x1000 µm slit) and automatically compared with the Rruff database [99].

## Supporting Information

Figure S1 Sketch section with evidence of the late Mousterian (A11-A5), Uluzzian (A4-A3) and the earliest Aurignacian layers (A2), with variable content in archaeological remains (increasing from light gray to dark gray and black). Center below, a section drawn 0,6m east of the main one (by M. Cremaschi & M. Peresani, redrawn by S. Muratori).
(TIF)

Figure S2 Map of Fumane Cave with the excavated area of A9 unit indicated in gray.
(JPG)

Figure S3 Three views of the context where the shell was found in unit A9 in the rear of the cave. Above, the entrance of cave during the fieldwork. Below, unit A9 in square 147 with flakes and bones embedded in dark sediment.
(PDF)

Table S1 Available radiometric dates for the Mousterian units of Fumane Cave (data from [40,41]).
(XLS)
