# Supplementary figures and images for: An Ochered Fossil Marine Shell From the Mousterian of Fumane Cave, Italy
Source: PLoS One. 2013 Jul 10;8(7):e68572. doi: 10.1371/journal.pone.0068572 (PMC3707824; doi:10.1371/journal.pone.0068572)

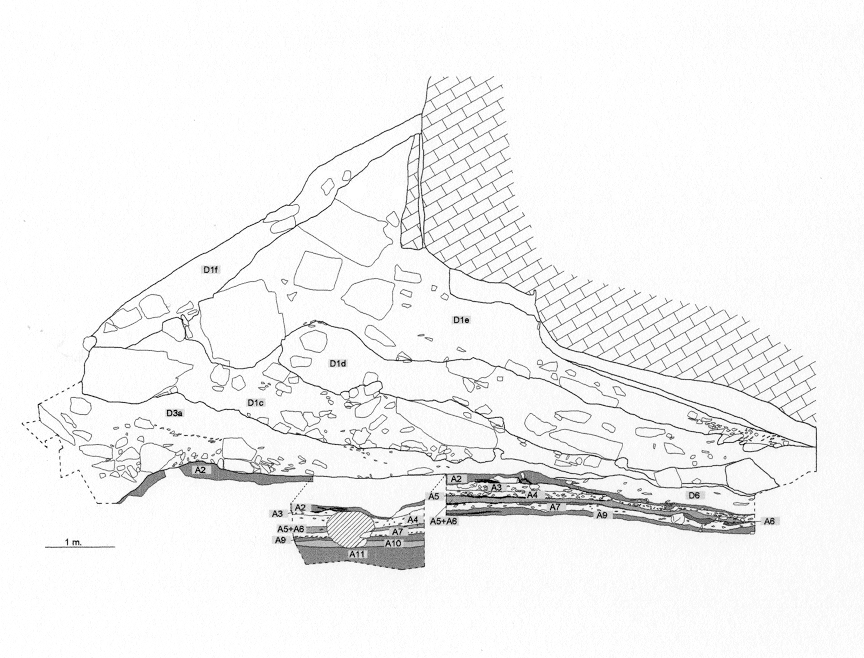

Supplement: Figure S1 — Center below, a section drawn 0,6m east of the main one (by M. Cremaschi & M. Peresani, redrawn by S. Muratori). (TIF) [file pone.0068572.s001.tif]

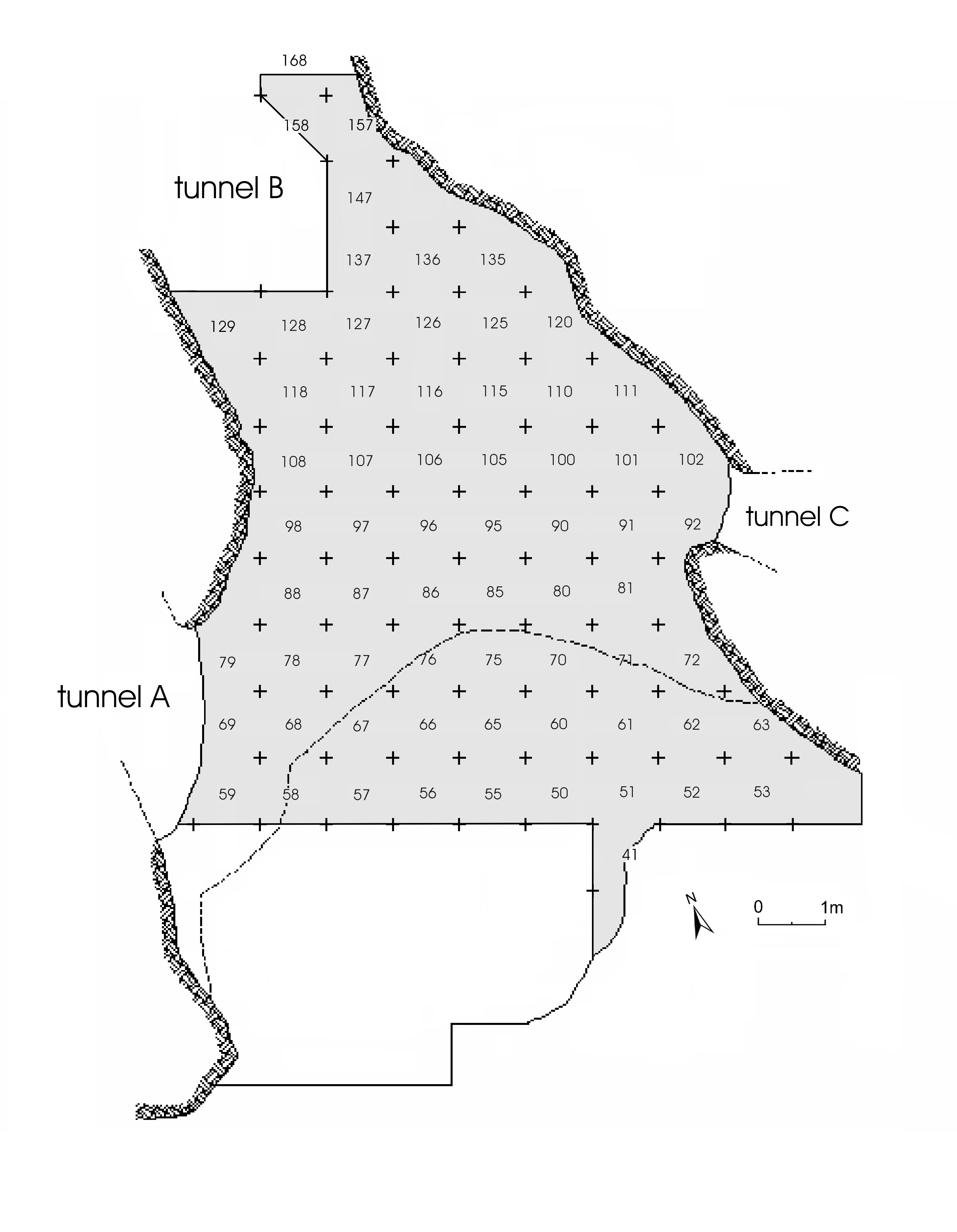

Supplement: Figure S2 — Map of Fumane Cave with the excavated area of A9 unit indicated in gray. (JPG) [file pone.0068572.s002.jpg]

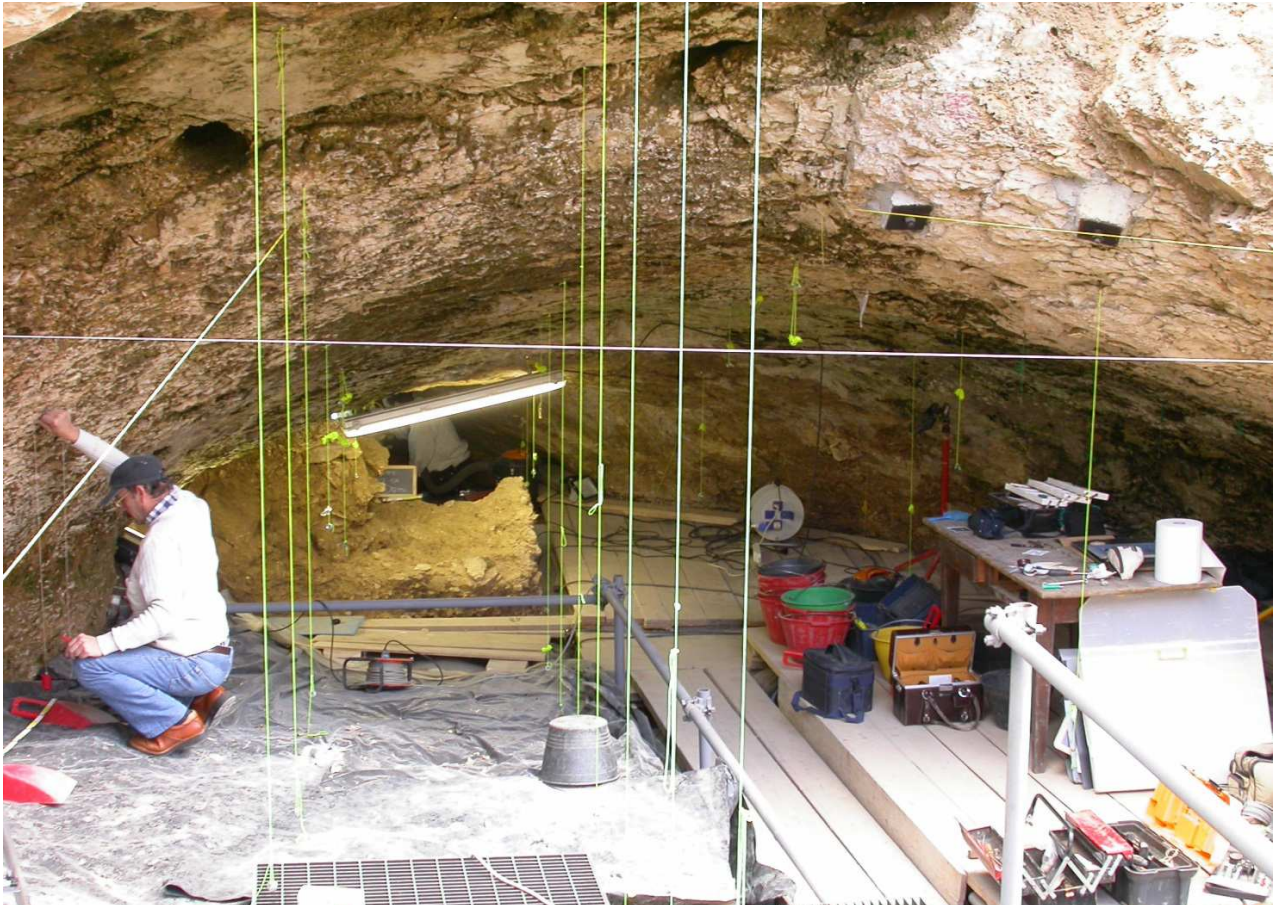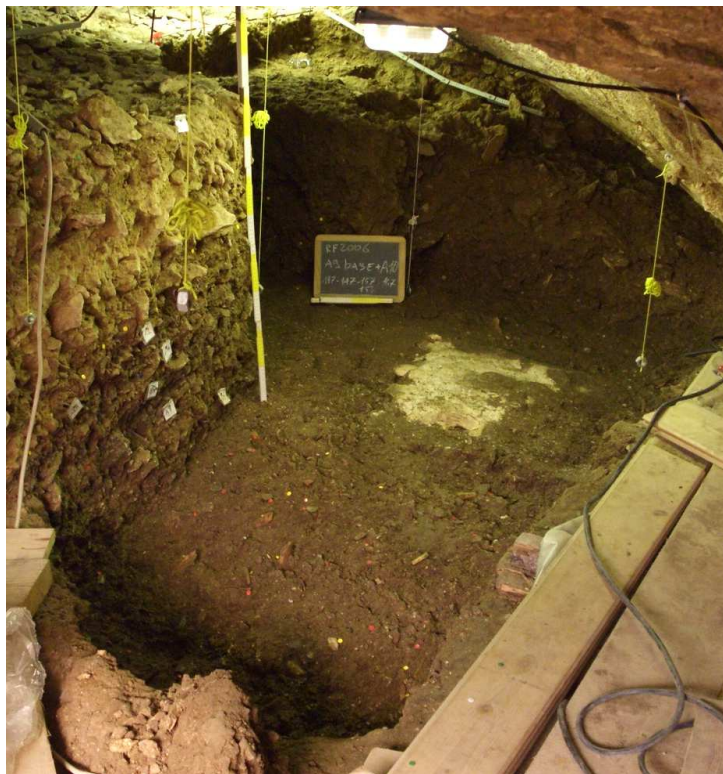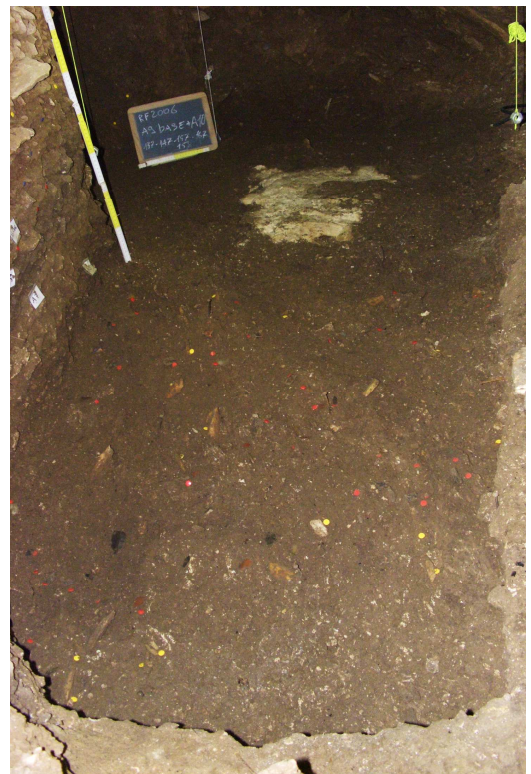

Supplement: Figure S3 — Three views of the context where the shell was found in unit A9 in the rear of the cave. Above, the entrance of cave during the fieldwork. Below, unit A9 in square 147 with flakes and bones embedded in dark sediment. (PDF) [file pone.0068572.s003.pdf]
